# Overview on population screening for carriers with germline mutations in mismatch repair (MMR) genes in China

**DOI:** 10.1186/s13053-021-00182-1

**Published:** 2021-05-01

**Authors:** Min Zhang, Tianhui Chen

**Affiliations:** 1 School of Public School, Hangzhou Medical College, Hangzhou, China; 2 Department of Cancer Prevention/Experimental Research Center, Cancer Hospital, Institute of Basic Medicine and Cancer (IBMC), University of Chinese Academy of Sciences (Zhejiang Cancer Hospital), Chinese Academy of Sciences, Hangzhou, China

**Keywords:** Population screening, Mismatch repair (MMR) genes, Lynch syndrome

## Abstract

DNA mismatch repair (MMR) genes play an important role in maintaining genome stability. Germline mutations in MMR genes disrupt the mismatch repair function and cause genome instability. Carriers with MMR germline mutations are more likely to have MMR deficiency and microsatellite instability (MSI) than non-carriers and are prone to develop colorectal cancer (CRC) and extracolorectal malignancies, known as Lynch syndrome (LS). MMR gene testing for suspected mutation carriers is a reliable method to identify the mutation types and to discover mutation carriers. Given that carriers of MMR germline mutations have a higher risk of LS-related cancers (LS-RC) and a younger age at onset than non-carriers, early surveillance and regular screening of relevant organs of carriers are very important for early detection of related cancers. This review mainly focuses on the general status of MMR carriers, the approaches for early detection and screening, and the surveillance of MMR mutation carriers in China. Population screening of MMR germline mutation carriers in China will be helpful for early detection, early diagnosis and treatment of MMR mutation carriers, which may improve the 5-year survival, and reduce mortality and incidence rate in the long term.

## Introduction

DNA mismatch repair (MMR) genes play an important role in maintaining genome stability. Germline mutations in MMR genes disrupt their mismatch repair function, cause genome instability and lead to increased cancer risk in the mutation carriers as represented by Lynch syndrome (LS) or hereditary nonpolyposis colorectal cancer (HNPCC) [1]. MMR genes, such as mutL homolog 1 (*MLH1*), MutS homolog 2 (*MSH2*), MutS homolog 6 (*MSH6*) and postmeiotic segregation increased 2 (*PMS2*), express MMR proteins, which are nucleic acid hydrolases that hydrolyze mismatched bases in the process of DNA replication to make DNA replication accurate. However, germline mutations in MMR genes cause MMR protein deficiencies, which lead to mismatch repair during replication, resulting in microsatellite instability (MSI) and tumorigenesis. A large-scale comprehensive screening found that 540 MMR variants were identified in the Chinese population, and the pathogenic/likely pathogenic carrier rate was 1.6 %. More than 90 % of the variants only existed in Chinese ethnicity, showing an ethnic-specific nature [1]. In 2019, a new *MLH1* c.1896 + 5G > A germline mutation was found in the Chinese LS-related lung and gastric cancer [2]. MMR mutation carriers susceptibly develop colorectal cancer (CRC) and other extracolorectal malignant tumors. It is a hereditary disease with an autosomal dominant inheritance. The incidence of LS in MMR mutation carriers is significantly higher than that in the general population, and the age at onset tends to be younger [3]. Nevertheless, LS patients usually have a decent clinical prognosis if they could be detected early and treated timely. Therefore, population screening for carries with MMR mutation has important clinical implications for LS-related cancers (LS-RC), such as improving 5-year survival, reducing the mortality, and reducing the incidence in the long-term. For example, regular colonoscopic screening for MMR mutation carriers could reduce CRC morbidity and mortality by 65–70 % [4].

## LS-related cancers and MMR mutations in China

Generally, LS-CRC located predominantly at proximal colon accounted for 2.7 % of total CRC in China [4], and the average age at onset was under 50 years old among Chinese population [5, 6]. Gastric cancer was mainly extracolorectal cancer in Chinese families [7, 8], while endometrial cancer was the most common extracolorectal malignancy in Western countries [3]. In neighboring Japan, LS-related gastric cancer was also the most common extracolorectal tumor, with a standardized incidence ratio estimated at 20.2 [9]. Others reported that the LS-related liver cancer in Southern China [10] and LS-related lung cancer in Northeast China [11] were more frequent than endometrial cancer. However, the incidence of LS-related endometrial cancer had been increasing rapidly in China recently [12–14]. *MLH1* and *MSH2* are the most common germline mutations reported by clinical research, accounting for about 90 % of all MMR gene mutations [15]. 11 *MLH1* variants, such as c.1588_1590delTTC, c.1163_1164insT and c.265G > T, and 18 *MSH2* variants, such as c.943-1G > A and c.595T > C were the high-frequency MMR variants in China [1]. In neighboring Korea and Japan, *MLH1 and MSH2* was also the most common germline mutation [9, 16]. In Pakistan, pathogenic/likely pathogenic MLH1/MSH2 variants account for a large proportion of HNPCC/suspected HNPCC colorectal cancer, mainly including three recurrent variants (MLH1 c.1358dup and c.2041G > A; MSH2 c.943-1G > C) [17]. Moreover, missense mutation was the most common mutation type in Chinese LS [18, 19]. Although mutations in MMR genes do not mean that cancer will definitely occur, the related cancer risk in MMR mutation carriers is significantly higher than that of the general population. The average cumulative risk of LS-RC in *MLH1* and *MSH2* mutation carriers was 9.7 %, 38.9 %, 69.5 %, 92.4 %, CRC was 9.7 %, 36.4 %, 66.7 %, 81.3 %, and gastric cancer was 0, 1.4 %, 6.1 %, 29.6 %, at age 30, 40, 50, 60 years old, respectively [20]. By the age at 70 years, the cumulative risk of *MLH1* and *MSH2* mutation carriers for all LS-RC would be 93.8 % [15], which was higher than western countries. In addition, the mean age of CRC diagnosis in *MLH1* and *MSH2* mutation carriers was lower than that in *MSH6* and *PMS2* [16, 21]. The mutation rate and characteristics of *PMS2* were rare in China. Compared with *MLH1* mutation, patients with *MSH2* mutation were more likely to develop synchronous and metachronous colon cancers [22]. Moreover, *PMS2*-deficient CRC was more common in the right colon and less in the rectum with poorly differentiated carcinoma [23]. LS-related endometrial cancer accounted for 4.78-5.4 % of total Chinese endometrial cancer, and *MSH6* was likely to be a Chinese founder mutation for endometrial cancer [10, 24]. The distribution of MMR gene pathogenic mutation in LS-CRC in China was as follows: *MLH1* 39.1 %, *MSH2* 33.9 %, *MSH6* 12.2 %, *PMS2* 9.6 % [4]. According to the studies from non-Asian population, *MSH2* and *MSH6* are the main susceptibility genes for ovarian cancer [25], *MSH2* for urinary tract cancer [21], and *PMS2* for breast cancer [26]. These LS-RC have not been fully studied in Asian population.

## Screening and diagnosing criteria and methods

Amsterdam II criteria, revised Bethesda guideline, and Japanese criteria are common clinical diagnostic criteria for LS. Amsterdam criteria had lower sensitivity and higher specificity, while the revised Bethesda guideline had higher sensitivity and lower specificity [27]. In 2003, the Chinese LS screening criteria, also known as Fudan criteria, were proposed, which requests to include the below items1-2 and at least one item among items 3–5: (1) There are at least 2 cases of CRC confirmed by histology in the family; (2) Two of them are first-degree relatives (parents and children or siblings); (3) At least one patient with multiple CRC (including adenoma); (4) At least one case of CRC occurred before 50 years old; (5) At least one case in the family had LS-related extracolorectal malignancies (including gastric, endometrial, small bowel, ureteral, renal pelvis, ovarian, and hepatobiliary cancer). Actually, the sensitivity and accuracy of the Chinese criteria was slightly higher compared to Amsterdam criteria. According to the findings from Fudan University Shanghai Cancer Center (China), for the identification of Chinese hereditary nonpolyposis colorectal cancer (HNPCC), the sensitivity, specificity and Youden index for Fudan criteria were slightly higher compared to those for Amsterdam criteria, i.e., 75 %, 58 and 33 %, respectively for Fudan criteria *versus* 50 %, 81 and 31 %, respectively for the Amsterdam criteria [22]. Furthermore, other investigations also confirmed that Amsterdam criteria may not be applicable to the diagnosis of HNPCC for Asian population [28]. However, most MMR mutation carriers were not easily identified by these clinical criteria [13]. Therefore, molecular screening strategies have greatly improved the diagnostic accuracy of LS. MMR gene testing is considered as the gold method for finding out MMR mutation carriers worldwide [15]. However, this high-cost technology has not yet been fully transformed into clinical applications in China. Currently, cheap accessible immunohistochemistry (IHC) technology has been widely used in China to detect MMR protein expressions [29]. *MLH1* missense variant caused the defects in *MLH1* and *PMS2* proteins expressions [19], and *MSH6* mutation and rarely *MSH2* mutation resulted in loss of *MSH6* protein expression [23]. Studies had shown that the sensitivity and specificity of IHC to detect MMR protein mutations were 66.7-90 % and 89-97.3 %, respectively [13, 18, 23]. MSI testing is another common screening and diagnostic method. The sensitivity and specificity of MSI testing in the diagnosis of LS-related endometrial cancer were 100 and 89.9 %, respectively [13]. Since approximately 15 % of sporadic cancer patients had MSI in tumor tissues, MSI testing usually combined with other methods to reduce the diagnosis errors [13, 24]. The sensitivity and specificity of combined IHC and MSI detection approach reached 100 and 72.4 %, respectively for screening LS-related endometrial cancer in Chinese patients, though only 111 patients were evaluated in this study [13]. Another study including 738 CRC patients found that IHC and MSI testing were only moderately concordant in assessing MMR deficiency and MSI-high status [31], suggesting a combination of the two approaches to provide reliable data.

Generally, protein deficiency cases are identified using IHC (for BRAF V600E mutation) and/or *MLH1* promoter methylation (for *MLH1* mutation). The BRAF V600E mutation rate for *MLH1*-deficient CRC in Chinese population was significantly lower compared to Western population [4]. LS patients and mutation carriers were diagnosed by MMR gene testing from *MLH1*-deficient cases with *MLH1* unmethylation, *MLH1*-deficient cases with wild-type BRAF CRC, and other protein deficiency cases [4]. Additionally, *MLH1* promoter-methylated CRC was also detected among individuals with family history of cancer and *MLH1* splicing mutant [4, 32], indicating that family history of LS-RC is an important factor to avoid missing suspected case. Next-generation sequencing (NGS) and multiplex ligation-dependent probe amplification (MLPA) are also promising approaches for gene testing, which can provide better and faster gene characteristics for cancer patients and high-risk individuals [6, 33, 34].

## Follow‐up surveillance and regular screening for MMR mutation carriers

Individuals with *MLH1* or *MSH2* gene mutation are generally advised to undergo colonoscopy every 1–2 years from age at 20–25 years or 5 years earlier than the youngest CRC patient in the family [15, 33, 35]. For *MSH6* or *PMS2* mutation carriers, some studies recommended colonoscopy surveillance should be started in later years [36, 37] (Table 1). Among mutation carriers, the colonoscopy surveillance group could detect more early cancers compared to the non-surveillance group (70.0 % vs. 36.5 %) [15]. Generally, CRC risk in mutation carriers increased with age, and male carriers with *MLH1* and *MSH2* had a higher risk of LS-CRC compared to female carriers at age of 70 years [37]. Therefore, the counseling and management strategies for carriers with different MMR mutation shall be different [37]. In order to monitor and screen for endometrial and ovarian cancers, female mutation carriers were recommended to perform annually gynecological examination, pelvic ultrasound and endometrial biopsy from the age of 30–35 years [5, 28, 35]. Currently, preventive hysterectomy and oophorectomy are recommended for female carriers over 40 years or after childbirth [33]. Since gynecological screening has no effect on the increased risk of ovarian cancer reported in the literature, prophylactic oophorectomy is also recommended for female MMR mutation carriers over 35 years [35, 37]. It is also recommended to perform upper gastrointestinal endoscopy with gastric biopsy every 1–3 years starting from 30 to 35 years old and to remove helicobacter pylori completely [35]. Currently, surveillance guidelines for small intestine, urinary tract, lung, pancreas and brain cancers are still lacking. The risk, age at diagnosis, regular screening method, starting surveillance age, and survival of MMR mutation carriers, are presented in Table 1.

**Table 1:** The risk, age at diagnosis, regular screening method, starting age for surveillance, and survival of MMR mutation carriers

LS-RC	MMR mutation rate (%)	Cumulative risk at age 70 years (%)	Diagnosis age (years)	Screening method	Starting age for surveillance	5-year survival	Reference
CRC	*MLH1*:37.5-55.6 *MSH2*:33.8-50.0 *MSH6*:12.2-41.4 *PMS2*:9.6–57.9	Mean:80 *MLH1*: 52-81.7 MLH2: 52-93.1 *MSH6*: 10-22 *PMS2*: 15–20	Mean:45.7 Male:42 Female:47 *MLH1*:44 *MSH2*:43 *MSH6*:52 *PMS2*:46	Colonoscopy every 1–2 years	*MLH1* and *MSH2* carriers: 20–25 years *MSH6* and *PMS2* carriers: 30–35 years	79.2–84.2	[4, 6, 7, 11, 15, 16, 19–24, 31, 32, 35–37]
Endometrial cancer	*MSH2*:50-66 *MLH1*:24-40 *MSH6*:10-13 *PMS2* 9.6	Mean:51 *MLH1*: 42 *MSH2*: 56 *MSH6*: 45	Mean: 49-49.7 *MLH1* or *MSH2*:48-62 *MSH6*:55 *PMS2*:49	Gnecological examination, pelvic ultrasound and endometrial biopsy every year. After giving birth to hysterectomy.	30–35	96.2	[3, 5, 12–14, 25, 35, 37]
Gastric cancer	NR	Male *MLH1* or *MSH2*>32.8 Female *MLH1* or *MSH2*>27.1	Mean: 56-58.5 *MLH1* or *MSH2*: 56 *MSH6*: 63 *PMS2*: 70–78	Gastroscopy every 3–5 years and remove Helicobacter pylori completely	30–35 or 40–45	61–64	[2, 8, 9, 21, 30, 35]
Lung cancer	NR	NR	68.5	Appropriate imaging examination screen and surveillance for carriers	NR	NR	[2, 11, 35]
Ovarian cancer	*MLH1*:38 MLH2:47	*MLH1* or *MSH2*: 11–24 *MSH6*:3-10 *PMS2*: 6	Mean: 45-46 *MLH1* or *MSH2*: 44 *PMS2*: 42	Vaginal ultrasound and CA125 detection, bilateral fallopian tube-ovarian resection after childbirth	30–35	52.5–59	[11, 33, 35, 37]
Small bowel cancer	NR	*MLH1* or *MSH2*: 3–6	*MLH1* or *MSH2*: 47-49 *MSH6*: 54 *PMS2*: 59	Upper gastrointestinal endoscopy every 3–5 years	30–35	NR	[28, 33]
Urinary tract cancer	NR	1-12.6	*MLH1* or *MSH2*: 54-60 *MSH6*: 65	Urinalysis and ultrasound per year	30–35	85–93	[22, 28, 33]
Pancreatic cancer	NR	3.68	NR	Endoscopic ultrasound and magnetic resonance cholangiopancreatography based on individual conditions	NR	<5	[28, 33]
Central nervous tumors	NR	1–3	*MLH1* or *MSH2*: 50 *PMS2*: 45	Routine physical examination every year	25–30	22	[28, 33]

*NR* not reported

## Conclusions and future perspectives

Germline mutations in MMR genes in Chinese population show an ethnic-specific nature. In China, 54.8 % of family members with Lynch syndrome carried MMR gene mutations [15]. Further investigations on population screening for carriers with MMR germline mutations in Chinese population are highly warranted, which may promote to prevent precisely, to detect and diagnose early, and to treat effectively for MMR mutation-related cancers. Eventually, population screening may increase 5-year survival, and reduce mortality and incidence in the long term for LS-RC.

## Data Availability

The articles used in this review are available from the corresponding author.

## Abbreviations

MMR: DNA mismatch repair
MLH1: MutL homolog 1
MSH2: MutS homolog 2
MSH6: MutS homolog 6
PMS2: Postmeiotic segregation increased 2
LS: Lynch syndrome
LS-RC: Lynch syndrome-related cancers
HNPCC: Hereditary nonpolyposis colorectal cancer
MSI: Microsatellite instability
CRC: Colorectal cancer
LS: Lynch syndrome
IHC: Immunohistochemistry
NGS: Next-generation sequencing
MLPA: Multiplex ligation-dependent probe amplification
