# Correction to: Overview on population screening for carriers with germline mutations in mismatch repair (MMR) genes in China

**DOI:** 10.1186/s13053-021-00184-z

**Published:** 2021-05-17

**Authors:** Min Zhang, Tianhui Chen

**Affiliations:** 1 School of Public Health, Hangzhou Medical College, Hangzhou, Zhejiang China; 2 Department of Cancer Prevention/Experimental Research Center, Cancer Hospital of the University of Chinese Academy of Sciences (Zhejiang Cancer Hospital), Institute of Basic Medicine and Cancer (IBMC), Chinese Academy of Sciences, Hangzhou, China

**Correction to: Hered Cancer Clin Pract 19, 26 (2021)**

**https://doi.org/10.1186/s13053-021-00182-1**

Following publication of the original article [1], a typesetting errors was identified.

1. The author affiliations were incorrect and have been updated in this correction article.
2. **LS-related cancers and MMR mutations in China**, paragraph 1, line 24, the sentence should read: In Pakistan, pathogenic/likely pathogenic MLH1/MSH2 variants account for a large proportion of HNPCC/suspected HNPCC colorectal cancer, mainly including three recurrent variants (MLH1 c.1358dup and c.2041G > A; MSH2 c.943-1G > C) [17].
3. New reference 17 should be: Rashid MU, Naeemi H, Muhammad N, Loya A, Lubiński J, Jakubowska A, et al. Prevalence and spectrum of MLH1, MSH2, and MSH6 pathogenic germline variants in Pakistani colorectal cancer patients. Hered Cancer Clin Pract. 2019;17: 29.
4. **Screening and diagnosing criteria and methods**, paragraph 1, line 21, "hereditary nonpolyposis colorectal cancer" should be deleted and the sentence should read: Chinese criteria was slightly higher compared to Amsterdam criteria. According to the findings from Fudan University Shanghai Cancer Center (China), for the identification of Chinese HNPCC …

The original article [1] has been corrected.
